# Factors Associated with Smoking Cessation in Underdeveloped Areas of Indonesia

**DOI:** 10.34172/jrhs.11518

**Published:** 2025-10-18

**Authors:** Muhamad Arif Musoddaq, Dwi Hapsari Tjandrarini, Felly Philipus Senewe, Alfons Maryono Letelay, Hadi Ashar, Budi Setyawati, Ning Sulistiyowati, Maxwell Landri Vers Malakauseya, Christiana Rialine Titaley

**Affiliations:** ^1^National Research and Innovation Agency, Republic of Indonesia, Bogor, West Java, Indonesia; ^2^Faculty of Medicine, Universitas Pattimura, Ambon, Maluku, Indonesia; ^3^Department of Public Health, Faculty of Medicine, Universitas Pattimura, Ambon, Maluku, Indonesia

**Keywords:** Cigarette smoking, Smoking cessation, Risk factors, Indonesia

## Abstract

**Background:**

Indonesia has one of the highest smoking rates globally. Smoking cessation is critical for reducing smoking-related diseases, particularly in areas with limited healthcare access. This study explored factors associated with smoking cessation in underdeveloped areas of Indonesia.

**Study Design:**

This study was conducted using a cross-sectional design.

**Methods:**

Data were obtained from the 2018 Indonesia Basic Health Research survey. We used information from 16,989 ever-smokers aged 10 years or older living in underdeveloped areas of Indonesia. Binary logistic regression analyses were performed to identify factors associated with smoking cessation.

**Results:**

Overall, 8.1% of ever-smokers in underdeveloped areas of Indonesia had stopped smoking cigarettes at the time of the survey. Increased odds of smoking cessation were were observed among respondents living in urban areas (aOR=1.50, 95% CI: 1.13-2.00), females (aOR=2.59, 95% CI: 1.85-3.62), aged over 45 years (aOR=2.60, 95% CI: 2.13-3.17), the unemployed or students (aOR=1.60, 95% CI: 1.24-2.01), and heads of households (aOR=1.84, 95% CI: 1.45-2.32). Non-daily smokers (aOR=6.84, 95% CI: 5.68-8.24) and those who started smoking before age 18 (aOR=1.34, 95% CI: 1.10-1.62) were more likely to have quit smoking.

**Conclusion:**

Public health interventions should focus on supporting younger populations, informal workers, and daily smokers in rural areas to improve cessation rates.

## Background

 Smoking is a major global health issue prevalent across numerous countries worldwide. As of 2024, about one in every five adults globally was a smoker.^1^ In 2023, smoking was directly responsible for over eight million deaths annually, with an additional 1.3 million deaths among non-smokers due to exposure to second-hand smoke.^2^ Smoking has long been recognized as a major risk factor for cardiovascular and respiratory diseases,^3^ cancers,^4^ and other severe health conditions.^5^ Researchers have reported that the majority of tobacco-related deaths occurred in low- and middle-income countries.^2^

 While smoking prevalence in the World Health Organization (WHO) South-East Asia Region dropped from 68.9% in 2000 to 43.7% in 2022, an estimated 411 million people remain addicted.^6^ In Indonesia, 33.5% (68.9 million) smoked in 2021,^7^ placing the country among the top 10 globally for smoking by 2022.^8^ The smoking rate decreased further to 28.6% in 2023,^9^ likely due to public health campaigns, higher tobacco taxes, and stricter regulations.^10^

 In 2012, the Indonesian government mandated that all cigarette producers and importers include health warnings on their products to highlight the dangers of smoking.^11^ Local governments were also encouraged to establish smoke-free areas as a measure to reduce smoking prevalence and promote cessation.^12^ In 2017, a national program was launched to promote a culture of healthy living, with initiatives designed to discourage smoking and prevent its initiation.^13^ In 2022, the Minister of Finance announced a 10% increase in excise tax on tobacco products for both 2023 and 2024, alongside a rise in the minimum retail price of cigarettes.^14^ While these measures have not fully curbed smoking, smoking rates among Indonesians have gradually declined.^15^

 A study from Indonesia showed that current smokers face a 1.48 times higher risk of all-cause mortality compared to non-smokers.^16^ Additionally, most smokers in Indonesia were reported to belong to lower socioeconomic groups,^11^ who already experience increased risks of disease, disability, and premature death.^17^ According to Indonesian Presidential Regulation Number 63, underdeveloped areas in Indonesia are defined by a low economic status, limited human resources, inadequate infrastructure, weak regional finances, poor accessibility, and distinctive regional characteristics.^18^ Consequently, communities in such areas are particularly vulnerable to health issues, and smoking further compounds the health burden.^18,19^ Consistent with this, studies have reported that individuals living in disadvantaged neighbourhoods are more likely to smoke.^20^

 Despite this evidence, there is a scarcity of research focused on smoking cessation in Indonesia’s underdeveloped regions. Most existing studies have examined smoking prevalence or its health outcomes, but little is known about the factors that facilitate or hinder quitting in these vulnerable populations. Underdeveloped areas are characterized not only by economic hardship and limited health infrastructure but also by unique social and cultural dynamics, including strong community ties, local norms around tobacco use, and limited exposure to cessation campaigns. Geographic isolation and poor accessibility further constrain access to healthcare and cessation services. These factors create smoking behaviors, and the ability to quit, distinct from those in more developed settings.

 Addressing this gap is crucial for designing targeted and context-specific interventions that are both feasible and culturally appropriate. Therefore, this study aimed to examine the factors associated with smoking cessation in populations living in underdeveloped areas of Indonesia. The findings can help inform tailored public health strategies and policies to reduce smoking prevalence and improve health outcomes in these high-risk settings.

## Materials and Methods

###  Study Design and Data Collection 

 This study used data derived from the 2018 Basic Health Research of Indonesia, a national-scale community-based survey conducted regularly by the National Institute of Health Research and Development, Ministry of Health, Republic of Indonesia.^21^ The 2018 survey was conducted from April to May 2018 using a cross-sectional design and included households across all 34 provinces, 416 districts, and 98 cities. Data were collected through interviews, physical measurements, and health examinations. A multistage systematic random sampling method was used, with census blocks serving as the primary sampling unit, followed by household selection within each block. This design ensured nationally representative estimates at both provincial and district/city levels. The 2018 survey achieved a response rate exceeding 95%, and rigorous quality control procedures, including enumerator training, field supervision, and double data entry, were implemented to maintain data accuracy and reliability. Key indicators included health services, health behaviours, environmental factors, biomedical data, and health status. A detailed explanation of this survey has been provided elsewhere.^21^

 The study population for this analysis consisted of ever-smokers aged 10 years or older, residing in underdeveloped areas of Indonesia who were interviewed during the 2018 Basic Health Research (62 districts located in 11 provinces). Ever-smokers were defined as individuals who had smoked at least once in their lifetime, regardless of whether they continued smoking or had quit. Underdeveloped areas were defined as districts that are less developed compared to other areas on a national scale in terms of community economy, human resources, facilities and infrastructure, regional financial capacity, accessibility, and regional characteristics.^18^ According to government classification, there were 112 underdeveloped districts/cities in Indonesia between 2015 and 2019, a figure that decreased to 62 in 2020–2024.^22^

 In total, data from 16,989 ever-smokers aged 10 years or older residing in underdeveloped districts across 11 provinces were analyzed. Variables included demographic characteristics, medical conditions, healthcare history, and health behaviors of the respondents. As sampling weights were applied in the analysis, reported totals may vary slightly across variables due to rounding.

###  Outcome Variable

 The outcome variable in this study was smoking cessation, defined as ever-smokers who had not smoked in the 30 days preceding the survey.^21^ Respondents were classified into two groups: *smoking cessation* (those who had stopped smoking for at least 30 days) and *current smokers* (those who reported smoking daily or occasionally at the time of the survey, or who had quit less than 30 days before data collection).

###  Potential Predictors 

 Potential predictors of smoking cessation were categorised into three domains: type of residence (urban/rural), individual socio-demographic and household characteristics, and smoking exposure. The variables in the individual socio-demographic and household characteristics included sex (male/female), age group (10–45 years/ > 45 years), education level (high/low), occupation (informal workers/unemployed or students/formal workers), marital status (unmarried/married), and household status (member/head of household). The respondent’s education level was classified as high if they had completed at least senior high school and low if they had only completed junior high school or lower. Informal workers included entrepreneurs, farmers, fishermen, labourers, and those in similar roles, while formal workers encompassed those employed in formal sectors, such as government or private employees, members of the armed forces, and police officers. Single marital status included respondents who had never married or were divorced.

 In the smoking exposure group, several variables were included: presence of other ever-smokers in the household (yes/no), smoking frequency (daily/non-daily smoker), age of first smoking experience ( < 18 years/ ≥ 18 years), and consumption of different cigarette types, including clove cigarettes (yes/no), white (non-clove) cigarettes (yes/no), and self-rolled cigarettes (yes/no).

###  Data Analysis 

 In the first stage, a descriptive analysis was conducted to outline each variable used in this study. This was followed by a bivariable analysis to examine the distribution of each potential predictor by smoking status. Subsequently, logistic regression analysis was performed to identify the association between contributing factors and smoking cessation. Univariate logistic regression was used to assess the relationship between each potential predictor and smoking cessation status without adjustment for other predictors. In the final stage, multivariable logistic regression was conducted to evaluate the association between all potential predictors and smoking cessation status, controlling for the effects of covariates. Results of the logistic regression analyses are presented as odds ratios (ORs) with 95% confidence intervals (CIs). Data were analyzed using IBM SPSS Statistics version 21 for Windows.

## Results

**Figure 1:**
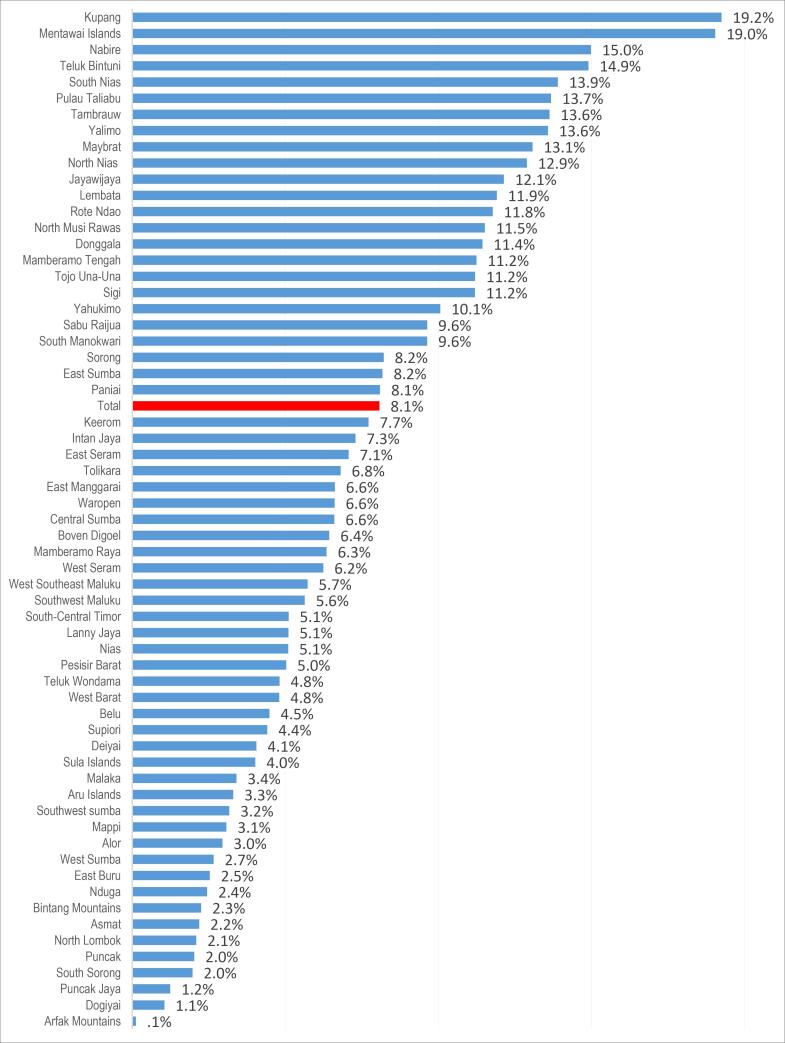
Percentage of smoking cessation in underdeveloped areas of Indonesia, Basic Health Research 2018

 Our analysis showed that of the total 16,989 ever-smokers living in underdeveloped areas of Indonesia, only 8.1% had stopped smoking cigarettes at the time of the survey. Smoking cessation rates varied significantly across districts, ranging from 0.1% in the Arfak Mountains District of Papua Province to 19.2% in the Kupang District of East Nusa Tenggara Province (Figure 1).

Table 1 presents the characteristics of all ever-smokers included in this analysis. The majority were male (95.1%, n = 16,151), under 45 years (72.3%, n = 12,284), had a low education level (70.3%, n = 11,937), and were employed in informal occupations (76.3%, n = 12,963). A higher proportion of ever-smokers were married (71.7%, n = 12,176), heads of households (64.7%, n = 10,993), and residents of rural areas (87.2%, n = 14,817). Among ever-smokers, 73.2% (n = 12,443) reported smoking daily, and 59.6% (n = 7,750) had started smoking before the age of 18. Regarding cigarette type, 75.9% (n = 12,897) consumed clove cigarettes, 37.2% (n = 6,321) smoked white (non-clove) cigarettes, and 29.1% (n = 4,948) smoked self-rolled cigarettes (Table 1).

**Table 1:** Characteristics of ever-smokers in underdeveloped areas in Indonesia, Basic Health Research 2018

**Variables**	**Number**	**Percent **
Type of residence		
Rural	14,817	87.2
Urban	2,171	12.8
Sex		
Male	16,151	95.1
Female	837	4.9
Age (y)		
≤ 45	12,284	72.3
> 45	4,704	27.7
Education Level		
Low	11,937	70.3
High	5,051	29.7
Occupation		
Informal workers	12,963	76.3
Unemployed/students	2,592	15.3
Formal workers	1,433	8.4
Marital status		
Unmarried (single/divorced)	4,812	28.3
Married	12,176	71.7
Status in the household		
Member of household	5,995	35.3
Head of household	10,993	64.7
Presence of smoker in the household		
Yes	7,095	41.8
No	9,893	58.2
Smoking frequency		
Daily smoker	12,443	73.2
Non-daily smoker	4,545	26.8
Age of first smoking (years)		
≥ 18	5,243	40.4
< 18	7,750	59.6
Consumption of clove cigarettes		
Yes	12,897	75.9
No	4,091	24.1
Consumption of non-clove cigarettes		
Yes	6,321	37.2
No	10,667	62.8
Consumption of self-rolled cigarettes		
Yes	4,948	29.1
No	12,040	70.9

 The distribution of factors associated with smoking status is presented in Table 2. Smoking cessation was 1.52 times more common among former smokers living in urban areas than those in rural areas (OR = 1.52, 95% CI: 1.18-1.97; *P* = 0.001). Smoking cessation was also 1.99 times more common among females than males (OR = 1.99, 95% CI:1.55-2.56; *P* < 0.001). Compared with informal workers, unemployed individuals or students were more likely to quit smoking, as were formal workers, with odds of 1.65 (OR = 1.65, 95% CI: 1.37-2.00; *P* < 0.001) and 1.70 times (OR = 1.70, 95% CI: 1.32-2.18; *P* < 0.001), respectively. Furthermore, smoking cessation was more common among heads of households, who were about 1.36 times more likely to quit than other household members (OR = 1.36, 95% CI: 1.16-1.61; *P* < 0.001). Non-daily smokers were substantially more likely to quit, with odds approximately 5.53 times greater than those of daily smokers (OR = 5.53, 95% CI: 4.71-6.50; *P < 0.001*). No significant differences in the prevalence of smoking cessation were observed by education level, marital status, presence of other smokers in the household, age at first smoking, or type of cigarette consumed (*P* > 0.05), as depicted in Table 2.

**Table 2:** Distribution and association of characteristics of ever-smokers by smoking status in underdeveloped areas of Indonesia, Basic Health Research 2018

**Variables**	**Smoking**	**Smoking Cessation (%)**	**Number**	**OR (95% CI)**	***P*** **-value**
Type of residence					
Rural	92	8	14,818	1.00	
Urban	89	11	2,171	1.52 (1.18, 1.97)	0.001
Sex					
Male	92	8	16,151	1.00	
Female	86	14	838	1.99 (1.55, 2.56)	0.001
Age (y)					
≤ 45	94	6	12,285	1.00	
> 45	87	13	4,704	2.41 (2.07, 2.79)	0.001
Education level					
Low	92	8	11,938	1.00	
High	91	9	5,051	1.13 (0.95, 1.33)	0.163
Occupation					
Informal workers	93	7	12,964	1.00	
Unemployed/Students	89	11	2,592	1.65 (1.37, 2.00)	0.001
Formal Workers	89	11	1,432	1.70 (1.32, 2.18)	0.001
Marital Status					
Unmarried (single/divorced)	93	7	4,812	1.00	
Married	92	8	12,177	1.17 (0.99, 1.38)	0.071
Status in the household					
Member of household	93	7	5,996	1.00	
Head of household	91	9	10,993	1.36 (1.16, 1.61)	0.001
Presence of smoker in the household					
Yes	92	8	7,096	1.00	
No	92	8	9,893	1.06 (0.90, 1.25)	0.473
Smoking frequency					
Daily smoker	96	4	12,443	1.00	
Non-daily smoker	81	19	4,546	5.53 (4.71, 6.50)	0.001
Age of first smoker(year)					
≥ 18	91	9	5,244	1.00	
< 18	92	8	7,750	0.92 (0.78, 1.09)	0.330
Consumption of clove cigarettes					
Yes	92	8	12,897	1.00	
No	92	8	4,092	1.02 (0.86, 1.20)	0.863
Consumption of non-clove cigarettes (white)					
Yes	93	8	6,322	1.00	
No	92	8	10,667	1.12 (0.95, 1.33)	0.169
Consumption of self-rolled cigarettes					
Yes	93	7	4,949	1.00	
No	91	9	12,040	1.29 (1.08, 1.54)	0.065

*Note.* OR: Odds ratio; CI: Confidence interval.

Table 3 presents the results of a multivariable analysis of factors associated with smoking cessation among ever-smokers in underdeveloped areas of Indonesia. The likelihood of smoking cessation was higher among those residing in urban areas (aOR = 1.50, 95% CI: 1.13-2.00, *P* = 0.005) compared to those in rural areas. In terms of individual socio-demographic and household factors, the odds of smoking cessation were greater among female smokers (aOR = 2.59, 95% CI: 1.85-3.62, *P* < 0.001), respondents aged over 45 years (aOR = 2.60, 95% CI: 2.13-3.17, *P* < 0.001), and those who were unemployed or still in school (aOR = 1.60, 95% CI: 1.24-2.01, *P* < 0.001). Household heads were also more likely to quit smoking than other household members (aOR = 1.84, 95% CI: 1.45-2.32, *P* < 0.001).

**Table 3:** multivariable analysis of household member characteristics and recent smoking status in underdeveloped areas of Indonesia, Basic Health Research 2018

**Variables**	**Adjusted OR (95% CI)**	***P*** ** value**
Type of residence		
Rural	1.00	
Urban	1.50 (1.13, 2.00)	0.005
Sex		
Male	1.00	
Female	2.59 (1.85, 3.62)	0.001
Age (y)		
≤ 45	1.00	
> 45	2.60 (2.13, 3.17)	0.001
Education level		
Lower	1.00	
High	1.26 (1.02, 1.55)	0.031
Occupation		
Informal workers	1.00	
Unemployed/Students	1.60 (1.24, 2.06)	0.001
Formal workers	1.29 (0.93, 1.78)	0.128
Status in the household		
Member of household	1.00	
Head of household	1.84 (1.45, 2.32)	0.001
Smoking frequency		
Daily	1.00	
Non-daily	6.84 (5.68, 8.24)	0.001
Age of first smoking (years)		
≥ 18	1.00	
< 18	1.34 (1.10, 1.62)	0.003

*Note.* OR: Odds ratio; CI: Confidence interval.

 Among factors related to smoking exposure, non-daily smokers exhibited significantly higher odds of quitting (aOR = 6.84, 95% CI: 5.68-8.24, *P* < 0.001). Furthermore, individuals who began smoking before the age of 18 had greater odds of cessation compared to those who started later (aOR = 1.34, 95% CI: 1.10-1.62, *P =*0.003).

## Discussion

 Our findings indicate a relatively high prevalence of smoking cessation in underdeveloped areas of Indonesia, which surpassed the national average. The likelihood of smoking cessation was significantly higher among females, individuals aged over 45, the unemployed or students, heads of households, urban residents, non-daily smokers, and those who started smoking before the age of 18. These findings highlight key demographic and behavioural factors that should be considered when tailoring smoking cessation interventions. By targeting these individuals, public health efforts could further accelerate cessation rates, thereby improving overall health outcomes and reducing the burden of smoking-related diseases in vulnerable populations, particularly those living in the underdeveloped areas of Indonesia.

 The relatively high smoking cessation rate observed in underdeveloped areas of Indonesia indicates that, despite having fewer resources, communities living in these areas may benefit from specific social and environmental factors that encourage smoking cessation. This is supported by findings from other studies showing increased cessation in Indonesia (31.9%), Mexico (16.9%), and China (15.9%) but decreased rates in Turkey (140.4%), Vietnam (43.1%), and Romania (62.4%) in 2024.^23^ Community dynamics and social norms in underdeveloped regions could play a significant role, as close-knit communities often experience stronger social pressure or undergo shifts in attitudes towards smoking.^24^ These changes, sometimes led by local leaders or supported by health initiatives, can have an immediate influence on individual behaviour. Additionally, economic pressures can also contribute, as limited financial resources in underdeveloped areas make cigarette purchases a significant burden.^25^ Rising tobacco prices and increased taxation can further motivate individuals to view smoking cessation as a practical way to improve household finances.^10^ Additionally, public health programmes, such as targeted health campaigns, may be more effective in some rural areas, possibly due to their focus on community involvement and the promotion of healthier lifestyles.^26^

 Our study identified the effect of environmental factors on smoking cessation in underdeveloped areas of Indonesia. Those living in urban areas were more likely to quit smoking, which is consistent with previous reports.^27^ Smoking cessation efforts have generally been more successful in urban areas than in rural regions.^28^ Urban areas typically offer greater access to health facilities and media, providing communities with increased exposure to health information and smoking cessation resources compared to those living in rural areas.^29^ A similar trend was reported in Bangladesh, showing an increased prevalence of cigarette smoking by 0.3% between 1990 and 2015.^30^ Furthermore, stricter implementation and enforcement of no-smoking policies in urban compared to rural areas may lead to higher cessation rates among urban residents.^27^

 At the individual level,females demonstrated a higher likelihood of quitting smoking, consistent with trends observed at the national level in Indonesia.^31^ Negative societal perceptions of women who smoke may serve as a stronger motivator for cessation among females compared to males.^32^ Globally, female smokers are often aware of such stigma, which may further encourage them to quit.^33^ Research has also reported that women are more likely to stop smoking when motivated by the desire to protect children from second-hand smoke.^34^

 Our study also confirmed that older smokers are more likely to quit than younger smokers, consistent with the findings in previous literature.^21,35^ Specifically, individuals aged over 45 years showed a greater likelihood of smoking cessation compared to younger adults. Older smokers face a higher risk of developing smoking-related diseases, including chronic obstructive pulmonary disease, cardiovascular disease, and various cancers.^36^ Awareness of these health risks often increases with age, leading to greater reductions in smoking among older individuals.^37^

 In this study, unemployed individuals and students were more likely to quit smoking compared to those in informal occupations.^21^ High cigarette prices and increased tobacco taxation may have reduced purchasing power among those with limited or no income.^38^ For these groups, the financial strain of maintaining a smoking habit encourages them to quit smoking.^39,40^

 Being the head of a household is associated with a higher likelihood of quitting smoking in the underdeveloped areas of Indonesia. Heads of households may have greater motivation to quit than other family members, possibly due to concerns about the health of household members, financial responsibilities, and religious values.^41^ They might have a strong cultural and moral responsibility to protect the health and welfare of their family members. Religious values may also play an important role, as many faith traditions in Indonesia emphasize self-discipline, care for one’s body, and setting a positive example for others.^42^ These factors collectively may motivate household heads to prioritize smoking cessation for both family well-being and their own health.

 Intriguingly, occasional or non-daily smokers are more likely to quit smoking than daily smokers in underdeveloped areas. Nicotine dependence appears to be a key factor enabling cessation in these regions.^43^ However, we also found that smokers who started smoking before the age of 18 were more likely to quit than those who initiated smoking at an older age. This finding contrasts with previous studies on nicotine dependence, suggesting that early initiation generally leads to greater difficulty in quitting due to prolonged nicotine exposure and stronger dependence.^44^ Several factors may explain this finding. Younger smokers may be more exposed to health education, school-based prevention programs, and peer support networks, which raise awareness of smoking risks and promote cessation. In addition, tobacco control measures, such as higher cigarette prices, pictorial health warnings, and stricter age restrictions, may have a stronger impact on younger individuals by limiting access and reducing social acceptance.^45^ Early starters may also experience health problems earlier or receive stronger encouragement from family, peers, or healthcare providers to quit.^38^

 Reducing cigarette smoking in Indonesia requires a comprehensive, multifaceted approach. One crucial intervention is the implementation of smoke-free zones across all districts and cities, which not only protects the public from second-hand smoke but also creates environments that discourage smoking by reducing its social acceptability. Evidence shows that such policies help lower smoking rates by shifting societal norms regarding smoking behavior.^25^ Expanding healthcare-based cessation interventions is equally essential. Training healthcare practitioners to offer cessation counselling and integrating brief, targeted support into all levels of the healthcare system can significantly increase quit attempts, as smokers are more likely to succeed with professional support.^24^ Ensuring that these services are accessible at all levels further enhances their reach and impact.

 Public education campaigns through mass media, social media, and community outreach also play a key role in reshaping societal attitudes.^24,32^ By raising awareness of the risks of smoking and the benefits of quitting, these campaigns support behavior change and help reduce smoking prevalence over time. Additionally, increasing cigarette prices through higher excise taxes is a proven strategy for reducing smoking, particularly among younger individuals and those with lower incomes, who are more sensitive to price changes.^10^ Research consistently shows that higher cigarette prices lead to fewer people starting to smoke, more quit attempts, and greater overall cessation success.^10,46^ Moreover, revenue generated from tobacco taxes can be reinvested into public health programmes, further supporting prevention and cessation efforts to ensure a sustainable, long-term impact.

 This study has several strengths. First, the use of a large dataset enhances both statistical power and the generalisability of the findings. Second, given the limited research on smoking cessation in underdeveloped areas, this study provides valuable insights for designing interventions that are contextually appropriate for these regions. However, several limitations must be acknowledged. The focus on underdeveloped areas may introduce potential selection bias, limiting the generalisability of findings to more developed settings. In addition, important variables such as access to cessation resources, availability of healthcare services, and mental health status were not measured and may represent unmeasured confounders. Reliance on self-reported data also introduces the risk of recall and social desirability bias, particularly in reporting cessation status, which may affect the accuracy of the results. Finally, the cross-sectional design limits the ability to establish causal relationships between predictors and outcomes.

**Highlights**

- Only 8.1% of ever-smokers in underdeveloped areas of Indonesia had quit smoking, which is higher than the national rate.
- Smoking cessation was linked to age, sex, status, residence, smoking pattern, and early start.
- Recommended actions include the establishment of smoke-free zones, the provision of cessation services, targeted educational campaigns, and the implementation of higher tobacco taxes.

## Conclusion

 This study highlights key demographic and behavioural factors associated with smoking cessation in underdeveloped areas of Indonesia, offering actionable insights for targeted interventions. Public health efforts should prioritize the establishment of smoke-free zones in both urban and rural districts, the training of healthcare providers to deliver cessation counselling, and the implementation of tailored educational campaigns that resonate with local communities. Specific groups identified as having higher cessation rates, including non-daily smokers and women, may benefit from more focused approaches, for example, early counselling for non-daily smokers to prevent entrenched dependence, and community- or maternal-health-based campaigns for women. In addition, policies that increase cigarette prices through excise taxes can further discourage initiation and support cessation among vulnerable populations. Future longitudinal studies are required to confirm causal relationships and to assess the long-term effectiveness of tailored interventions in reducing smoking prevalence in these underserved regions.

## Acknowledgments

 This study used data provided by the Ministry of Health, Republic of Indonesia. The authors are solely responsible for the study’s results.

## Competing Interests

 The authors have no competing interests associated with the material presented in this study.

## Ethical Approval

 This study was approved by the Health Research Ethics Committee of the National Institute of Health Research and Development (HREC-NIHRD), Ministry of Health, Republic of Indonesia (Approval No: LB.02.01/2/KE.024/2018). Written informed consent was obtained from all participants prior to data collection, and confidentiality of the information was strictly maintained throughout the survey.

## Funding

 The authors received no financial support for the research, authorship, and/or publication of this article.
